# Outcomes of Laparoscopic Partial Cystectomy of Bladder Endometriosis: A Report of 18 Thai Women

**DOI:** 10.1089/whr.2021.0003

**Published:** 2021-09-03

**Authors:** Srithean Lertvikool, Yada Tingthanatikul, Woradej Hongsakorn, Chartchai Srisombut, Katanyuta Nakpalat, Sawaek Weerakiet

**Affiliations:** ^1^ Department of Obstetrics and Gynaecology, Faculty of Medicine Ramathibodi Hospital, Mahidol University, Bangkok, Thailand.; ^2^ Women Health Centre, Chulabhorn Hospital, HRH Princess Chulabhorn College of Medical Science, Chulabhon Royal Academy, Bangkok, Thailand.; ^3^ Division of Obstetrics and Gynecology, Bangkok Hospital Udonthani, Udonthani, Thailand.

**Keywords:** bladder endometriosis, dysuria, laparoscopic partial cystectomy, pelvic pain, long-term follow-up

## Abstract

***Aim:*** To determine the outcomes of laparoscopic partial cystectomy (LPC) for bladder endometriosis (BE).

***Methods:*** This was a retrospective study using medical records of women who underwent LPC for BE between January 2009 and December 2017. Demographic characteristics, surgical findings, including surgical site and size of the bladder lesion, endometriosis at other locations, and pre- and postoperative hormonal treatment data were collected.

***Results:*** We analyzed data of 18 women with full-thickness BE. The patients had a mean age of 34 (range, 26–45) years and body mass index of 21.6 (range, 16.1–25) kg/m^2^. All women had dysmenorrhea. Other symptoms noted include dysuria, gross hematuria, and infertility. BE with a mean diameter of 2.7 cm (range, 1–5) was most commonly found at the posterior wall of the bladder (94.4%). Peritoneal endometriosis (94.4%), endometrioma (33.3%), and deep endometriotic nodules (22.2%) in the posterior compartment were also found. No surgical complications were observed. Postoperative hormonal treatment was administered to 14 (77.8%) patients. All symptoms improved after the surgery. No recurrence was found after 30 (range, 12–74) months of follow-up.

***Conclusion:*** LPC is an effective treatment option for BE.

## Introduction

Endometriosis is a common gynecological problem, defined as the presence of endometrial-like tissue outside the uterus, causing inflammation and pain.^1–3^ There are three forms of endometriosis, namely, superficial peritoneal endometriosis, endometrioma, and deep endometriosis (DE). DE is defined as *a* ≥ 5 mm endometrial tissue penetrating the peritoneum^4^ or newly defined adenomyosis externa, which typically includes multifocal lesions involving several structures and organs such as the rectum, rectosigmoid, sigmoid, or vesicouterine folds.^5^ Urinary tract endometriosis (UTE) is present in ∼1% of all women with endometriosis^6^ and in ∼52.6% of women with DE^7^; however, ∼50% of women with UTE may be asymptomatic.^8,9^

Bladder endometriosis (BE) is the most common form of endometriosis involving the urinary tract and occurring in 85% of women with UTE.^10^ Clinically, there are differences in symptoms depending on the location and size of the lesions.^8,9,11^ Women with BE may present with pelvic pain, dysuria, urination disorders, and hematuria.^10^ Some women with BE are asymptomatic and diagnosed incidentally during operative procedures.^10^

Hormonal treatments, oral contraceptive pills (OCP), and progestins are prescribed as first-line treatments to reduce the pain and urinary symptoms. These medicines can successfully suppress symptoms and reduce the size of the BE.^12^ However, recurrence of symptoms and nodules are noted after the therapy is discontinued.

Surgical treatment *via* excision of the nodules reduces the symptoms and the risk of recurrence is reduced due to the complete removal of the pathologic lesion. The transurethral resection (TUR) of bladder nodules and partial cystectomy of the bladder have been proposed to treat BE. Since the disease develops from the outside (serosa) to the inside (bladder mucosa), TUR cannot completely excise the mass resulting in a high risk of recurrence, and attempts to remove the entire nodule increase the risk of bladder perforation.^12^

Partial cystectomy of the bladder can be performed by laparotomy or laparoscopy. Laparoscopic partial cystectomy (LPC) has been performed by trained specialists^12,13^ worldwide. Recently, there have been reports of LPC of the bladder. Most studies, however, have been reported in Europe and the United States,^14–18^ including a recent large population study.^19^ A few case reports and case series of LPC have been reported by Asian gynecologists and urologists.^20,21^ The best surgical approach to DE remains unclear.^1^ Therefore, we conducted a retrospective study to show the outcomes of LPC of BE in Thai women.

## Materials and Methods

Between January 2009 and December 2017, 22 women underwent laparoscopic surgery, and the nodules were pathologically diagnosed with BE in the Endoscopic Unit, Department of Obstetrics and Gynecology, Faculty of Medicine Ramathibodi Hospital, Thailand. Electronic medical records were reviewed. We collected the following patient information: age, body mass index (BMI), parity, symptoms, types of work-up, surgical site findings, size and type of lesions, pre- and postoperative hormonal treatments, and the duration of follow-up. Only women who had a full-thickness BE were included in this study. Women whose endometriotic lesions did not involve the bladder mucosa were excluded. Clinical Trial Registration Number: MURA2018/183.

LPC was performed with a four-port laparoscopic technique under general anesthesia. The patients were placed in a lithotomy position before the procedure. LCP was performed as follows: a cystoscopy and bilateral catheterization of the ureters if the mass was close to the ureteric orifices; dissection of the vesicouterine space; bladder resection and complete removal of the BE by resecting through the healthy tissue around the nodule; suturing of the bladder defect in two layers, namely the mucosal layer using a Vicryl 3-0 suture and the seromuscular layer with a Vicryl 2-0 suture; and filling the bladder with diluted methylene blue in normal saline after suturing to ensure watertight closure.

After the surgery, a Foley catheter was retained for 7–10 days and a ureteric catheter for 2 weeks. Ceftriaxone 2 g was administered preoperatively to all the patients. Other surgical procedures for treating accompanying findings such as myoma, adenomyosis, endometrioma, and peritoneal endometriosis were also performed. The operations included excision of the peritoneal endometriotic lesions, ovarian cystectomy, DE nodule resection, hysterectomy, and myomectomy. Postoperative follow-up involved the following: checking for any abnormal symptoms such as pelvic pain, dysuria, hematuria, and dyschezia; conducting pain assessment tests (visual analog scale 0–10); and performing a pelvic examination and an ultrasonography scan for symptomatic patients.

The study was approved by the Ethics Clearance Committee on Human Rights Related to Research Involving Human Subjects, Faculty of Medicine Ramathibodi Hospital, Mahidol University, Thailand.

### Statistical analysis

Descriptive statistics were used for statistical analysis. The data are presented as mean (range) and percentages.

## Results

There were 22 women with BE in our database. Four women were excluded because their lesions did not involve the bladder mucosa. The clinical characteristics of the remaining 18 women are presented in Table 1. The mean age and BMI were 34 (range, 26–45) years and 21.6 (range, 16.1–25) kg/m^2^, respectively. Three women (16.7%) were parous.

**Table 1.** Summary of Characteristics, Operative Findings, and Operations

Pts	Age	BMI	Parity	Symptoms	Site	Size	Other endometriosis locations	Operations
1	40	19.2	0	Dysmenorrhea, dysuria, infertility	Post wall	3.7	Myo, Adeno, BE, DE, Perit	LPC, Myom, DE resect, cysto
2	33	19.8	2	Dysmenorrhea	Post wall	1	Adeno, OV, BE	LPC, TLH, OC, cysto
3	26	20.2	0	Dysmenorrhea, infertility	Post wall	4	BE, Perit	LPC, PE excise, cysto
4	30	20.9	0	Dysmenorrhea	Post wall	3	BE, Perit	LPC, PE excise, cysto
5	29	24.1	0	Dysmenorrhea, infertility	Dome	1	BE, OV	LPC, OC
6	45	18.2	0	Dysmenorrhea, dysuria	Post wall	2	BE, Perit	LPC, PE excise
7	30	19	0	Dysmenorrhea, infertility	Ant wall	1	BE, OV, Perit	LPC, OC, PE excise
8	32	31	0	Dysmenorrhea, dysuria, infertility	Post wall	2	BE, OV	LPC, OC, cysto & DJ stent
9	43	19.1	2	Dysmenorrhea, dysuria, hematuria	Post wall	5	BE	LPC, TLH, cysto & DJ stent
10	37	22.3	1	Dysmenorrhea, dysuria, hematuria	Post wall	3	BE, Perit	LPC, PE excise, cysto
11	30	16.1	0	Dysmenorrhea, dysuria	Post wall	1.5	BE, DE	LPC, DE resect, cysto
12	35	21.2	0	Dysmenorrhea, dysuria, hematuria, infertility	Post wall	5	BE, DE	LPC, DE resect, cysto
13	33	22	0	Dysmenorrhea, dysuria, hematuria, infertility	Post wall	2	BE, Perit	LPC, PE excise, cysto
14	38	24.2	0	Dysmenorrhea, dysuria	Post wall	3	BE, OV, Perit	LPC, OC, PE excise, cysto
15	32	25	0	Dysmenorrhea, dysuria, infertility	Post wall	4	Adeno, BE, OV	LPC, OC, PE excise, cysto
16	34	19.2	0	Dysmenorrhea, dysuria, infertility	Post wall	2	BE, Perit	LPC, PE excise, cysto
17	29	19.9	0	Dysmenorrhea, dysuria	Post wall	2.5	BE, Perit	LPC, PE excise, cysto
18	33	23	0	Dysmenorrhea, dysuria, infertility	Post wall	2	Myo, Adeno, BE, DE, Perit	LPC, DE resect, PE excise, cysto

Adeno, adenomyosis; BE, bladder endometriosis; BMI, body mass index; DE, deep endometriotic nodule at posterior compartment; LPC, laparoscopic partial cystectomy; Myo, myoma; OV, ovarian endometriosis; Perit, peritoneal endometriotic lesion; Pts, patients; TLH, total laparoscopic hysterectomy.

All women had dysmenorrhea. Symptomatic patients involved 13 (72.2%) women with dysuria, 4 (22.2%) women with gross hematuria, and 7 (38.9%) women with infertility. We also noted three (16.7%) women with microscopic hematuria. Preoperative BE diagnosis was verified in 12 patients (66.7%). Of these, the diagnosis was made by ultrasonography (*n* = 1), magnetic resonance imaging (MRI) (*n* = 3), and cystoscopy (*n* = 2). A combination of cystoscopy and MRI was used to diagnose four women, and two women were diagnosed by cystoscopy and ultrasonography. Therefore, six women were diagnosed incidentally during surgery with undetected lesions during preoperative ultrasonography.

BE was most commonly found at the posterior wall of the bladder (94.4%) and the dome (*n* = 1, 5.6%). These bladder nodules had a mean diameter of 2.7 cm (range, 1–5 cm). The mean Revised American Fertility Society (R-AFS) scores were 30.9 (range, 4–76). Endometriosis was found more than one region in 17 women (94.4%). Peritoneal endometriosis (94.4%) was the most common finding, followed by endometrioma (33.3%), and posterior DE nodule at the rectovaginal septum (22.2%). Three women (16.7%) had BE and minimal peritoneal endometriosis. An isolated BE was found in only one woman (5.6%) with the largest nodule of 5 cm in diameter.

Apart from LPC, other procedures were performed in the same setting, including excision of peritoneal endometriotic lesions (*n* = 18), ovarian cystectomy (*n* = 6), DE nodule resection (*n* = 4), total laparoscopic hysterectomy (TLH) (*n* = 2), and laparoscopic myomectomy (*n* = 1). During the operation, cystoscopy was performed in 15 women. Of these, 13 women had nodules ≥2 cm in diameter with a mean diameter of 2.9 cm. In 2 of these 15 women, Double-J stents were inserted because the nodule was very large in one woman and in the other the nodule was close to the ureteric orifice.

The mean duration of the entire operation and the blood loss volume were 220.8 (range, 90–540) minutes and 228.9 (range, 50–1400) mL, respectively. One woman underwent blood transfusion. All procedures were performed successfully by one of the three surgeons (S.L., Y.T., and C.S.) without any complications. One woman had a urinary tract infection for 5 days following the surgery and recovered after antibiotic therapy.

Preoperative hormonal treatment for 3 months was administered to 6 (33.3%) women to relieve the pain before surgery. The hormones administered included gonadotropin-releasing hormone agonists (GnRH-a, *n* = 3), depo-medroxyprogesterone (DMPA, *n* = 2), and OCP (*n* = 1).

Postoperative hormonal treatment was administered to 14 (77.8%) women, and in six women (42.9%), it was administered for 5–9 months. These women did not participate in the follow-up sessions. On phone follow-up for the six women, it was seen that none of the participants underwent subsequent hormonal treatments. Eight (57.1%) of 14 women were continuously followed up and they underwent hormonal treatments (DMPA, OCP, or dienogest) at the endoscopic unit after surgery. The mean follow-up time was 30 months (range, 12–74 months).

Pain relief was achieved in all women after the operation. No recurrence of BE was found in any of the women, including those followed up by phone.

## Discussion

We conducted this retrospective study to demonstrate the outcomes of an LPC for BE. No intraoperative and postoperative complications were observed for LPC in our study. Most importantly, no disease recurrence was noted after LPC of BE after a 30-month long-term follow-up.

The operative findings in our study were similar to those of other studies.^14,15,17^ Most women with BE had endometriosis in other locations, 94.4% in our study compared to about 63%–87% in other studies, including DE nodules at the posterior compartment and endometriomas.^14,15,17^ The site of the bladder nodule was mostly at the posterior wall, which was also seen in 88.9% of the women in this study, and in 53.3%–74.7% women in other studies.^15,17,22^

The underlying mechanism of BE pathogenesis includes implantation of regurgitated menstrual endometrial cells on the peritoneum of various organs in the pelvic cavity^12^ and the aforementioned findings support this hypothesis. Implantation of the endometrial cells induces inflammation, followed by the development of adhesions, and the formation of a fibrotic nodule.^12^ However, studies have shown that a successful outcome can be achieved after surgery.^23,24^ Because endometriosis in each woman was located at multiple regions, it affected the reproductive function (fecundity). However, it was difficult to measure this reproductive outcome in this condition, and hence the reproductive outcome was not measured in the current study.

Ultrasound is the first-line imaging modality for the diagnosis and exclusion of BE. In our study, six women were not diagnosed to have BE *via* preoperative ultrasonography, but BE was diagnosed intraoperatively. This may be due to the low index of suspicion for BE by the general gynecologists. This emphasizes that ultrasonography alone is an imperfect modality with a possibility of false negatives and false positives. False negative results may be related to the small size of the nodule that can easily be missed by the ultrasound.^9,25^

For the preoperative hormonal treatment, this study included women with full-thickness BE pathological reports that mainly had other endometriotic lesions and who were candidates for laparoscopic surgery. We did not include small lesions that might have been treated with hormonal treatment or lesions covering the ureteric orifice, because it was recommended as a contraindication for surgery by urologic surgeons. Therefore, only 1/3 of the patients in this study underwent preoperative hormonal treatment.

No complications, either intra- or postoperatively, were noted in our study. This suggests that LPC is associated with good outcomes. Similarly, previous literature reports rare incidences of complications, especially intraoperative complications.^14–16^ The postoperative vesicovaginal fistula was observed in ∼0.7% of cases.^15,19^ The factor involved in this complication may be the suturing technique for the repair of the defective bladder.

There are two types of suturing techniques: single- and double-layer sutures.^26^ Repairing a defective bladder after a partial cystectomy of BE can include any of these two types depending on the institution's protocols or surgeon's preference.^13,15,17,19,27^ The vesicovaginal fistula could have developed in two women in whom single- and double-layer sutures were used during the repair of the defective bladder.

However, the risk of this complication may be more prevalent when single-layer suture is used in women rather than double-layer. Some studies used a single-layer suturing technique in patients and they presented with a postoperative vesicovaginal fistula.^13,15,27^ Stopiglia et al.^27^ used a single-layer suture for repairing the defective bladder in 12 women without complications. In contrast, Chapron et al.^15^ used a single-layer suturing technique and reported vesicovaginal fistula complications in 2 out of 75 (2.7%) women with BE. In using double-layer sutures, vesicovaginal fistulas were observed in 2 (0.8%) out of 264 women.^19^ In addition, some authors reported no fistula complications in eight women after using the double-layer suturing technique.^22^

Similarly, in the current study, laparoscopic repair of the defective bladder with the double-layer suturing technique was also used in 18 women, resulting in no complications. Experienced urologists have suggested that the double-layer suturing technique should be performed as the standard practice for the repair of the defected bladder to prevent complications, particularly vesicovaginal fistulas.^28^

It is also important to determine whether ureteric catheterization should be performed in the LPC. This procedure is aimed at preventing injury to the ureter during partial cystectomy of the bladder. Fedele et al.^14^ and Chapron et al.^15^ performed ureteric catheterization in all patients before the laparoscopic surgery for BE. However, some authors did not perform ureteric catheterization because the size of all nodules was less than 2 cm in diameter.^22^ In another study, cystoscopy was performed preoperatively in some patients to confirm the diagnosis, but ureteric catheterization had not been performed in all patients despite the large size of the nodules.^17^ We agree with the recommendation of some authors^12^ that ureteric catheterization is optional.

The decision to perform this procedure is mainly based on the preference of surgeons during cystoscopy and the site of the endometriotic nodule. The recommendation has also suggested that if the caudal margin of the nodule is less than 2 cm from the interureteric ridge, catheterization is advised.^12^ In the current study, two women with BE underwent ureteric catheterization because one woman had a large nodule, and in the other, the margins of the nodules were close to the orifice of the ureter.

To improve the outcomes of the surgery, modern equipment or new techniques have been applied in LPC. Robot-assisted LPC has been used in women with BE.^29^ However, robot-assisted LPC had no advantages when compared to conventional LPC in terms of preventing intraoperative and early and late postoperative complications.^29^

More interesting is the combination of cystoscopic and laparoscopic approaches to manage BE. Most recently, case reports^20,30,31^ and case series^21,27,32^ where this combination approach was used have been reported. The procedure named as “modified light-to-light technique”^27^ or “See-Through technique”^21^ is the simultaneous performance of TUR and LPC. This procedure aims to overcome the limitations of each surgical technique toward a more beneficial outcome. It can precisely identify the lesion, including the edge of the infiltrated lesion. Therefore, it can completely remove the nodule without accidental removal of the healthy tissue.^12,21,27^

Performing this procedure requires two sets of equipment and teams that may not be easily available. Therefore, the medical utility and course-effectiveness of this procedure should be evaluated on a large scale.

Another satisfactory outcome of the surgery was that there was no recurrence of the disease in this study. This implied that radical excision of the nodules was obtained in all patients, similar to other studies.^13,15,22^ Chapron et al.^15^ reported that 75 women with BE who underwent LPC had no recurrence after 50.9 ± 44.6 months of follow-up. Other reports^13,22^ also showed the same results after a long follow-up period. Evidence from the most recent publications utilizing the See-Through technique demonstrated complete excision of the nodule with no reported recurrence of the nodule after 20–32 months of follow-up.^21,27^ Apart from the removal of the nodule, LPC can clinically relieve pain and urinary symptoms.^12,15^

However, after long-term follow-up, recurrence of pelvic pain^13^ and urinary symptoms^22^ were observed. More importantly, recurrence of nodules has been reported by some authors.^14,19^ Ceccaroni et al.^19^ reported a 2.3% recurrence rate in 264 women with BE after 12 months. This may suggest presence of residual nodules in some patients.

Postoperative medical treatment may prevent recurrence. Postoperative hormonal treatment has been used by several authors.^17^ Of the 31 women with no recurrence, 23 (74.2%) received hormonal treatment after partial cystectomy without mention of the type and duration of the treatment.^17^ Similarly, 14 (77.8%) of the 18 women in the current study received hormonal treatments of DMPA, OCP, or dienogest for various durations. Of the 14 women, 8 (57.1%) received hormonal treatment for a long period. No recurrence was observed in any of the women.

Although there has never been a study on the use of postoperative hormonal treatment in preventing recurrence in BE, it is used to treat women with other types of endometriosis such as endometrioma, and hence should be considered in the treatment of BE. Evidence has shown that OCP^33–35^ and levonorgestrel-releasing hormone intrauterine system (LNG-IUS)^35^ can decrease the risk of recurrence of ovarian endometriosis after long-term use (2–5 years).

Dienogest, a new progestin, has been used to treat endometriosis with effective results, and it is well tolerated for long-term use. The recurrence rate of endometrioma was 0%–3.8% after 24 months in women who were administered this medicine postoperatively, whereas the rate was 24% for expectant management.^36–39^

The main limitation of this study was the retrospective design, which is limited by the data available in the medical records. Further prospective studies with long-term systematic data collection for each aspect should be conducted.

In conclusion, this study involving a long-term follow-up of women with BE from Thailand supports LPC of the bladder as an effective treatment modality for BE. However, this should be performed by a multidisciplinary team of experienced doctors. Postoperative hormonal treatment may play a role in preventing the recurrence of the disease.

## Abbreviations Used

BE: bladder endometriosis
BMI: body mass index
DE: deep endometriosis
DMPA: depo-medroxyprogesterone
GnRH-a: gonadotropin-releasing hormone agonists
LNG-IUS: levonorgestrel-releasing hormone intrauterine system
LPC: laparoscopic partial cystectomy
MRI: magnetic resonance imaging
OCP: oral contraceptive pills
OV: ovarian endometriosis
R-AFS: Revised American Fertility Society
TLH: total laparoscopic hysterectomy
TUR: transurethral resection
UTE: urinary tract endometriosis
